# Detection and treatment of an aneurysma spurium of the arteria hepatica dextra after laparoscopic cholecystectomy

**DOI:** 10.1186/1471-230X-13-121

**Published:** 2013-07-25

**Authors:** Oliver Kirschberg, Andreas Scheding, Thomas Saers, Bernd Krakamp

**Affiliations:** 1 Department of Internal Medicine II, Witten/Herdecke University, Faculty of Health – School of Medicine, Campus Cologne-Merheim, Ostmerheimerstrasse 200, Cologne D-51109, Germany; 2 Department of Internal Medicine, HELIOS Klinik Wipperfuerth, Alte-Koelner-Strasse 9, Wipperfuerth D-51668, Germany; 3 Department of Internal Medicine, Gastroenterology and Diabetology, Vivantes Humboldt Klinikum, Am Nordgraben 2, Berlin D-13509, Germany

**Keywords:** Aneurysma spurium, Arteria hepatica, Upper gastrointestinal bleeding, Laparoscopic cholecystectomy

## Abstract

**Background:**

Massive upper gastrointestinal hemorrhage can be the dominant symptom of decompensated liver cirrhosis, varices and ulcerations in the upper gastrointestinal tract. Postoperative complications are known to lead to these bleedings. Commonly, emergency endoscopy will be performed. Here we report of a patient with extensive bleeding caused by an aneurysma spurium of the arteria hepatica dextra induced by a laparoscopic cholecystectomy. The condition was diagnosed by the Doppler ultrasound scan of the liver.

**Case presentation:**

Initially the source of the gastrointestinal bleeding was caused by an ulcus Dieulafoy in the jejunum which was stopped by clipping. Continous bleeding was observed and traced to a rare complication of a laparoscopic cholecystectomy due to a gallbladder empyema. After surgical intervention the patient developed an aneurysma spurium of the arteria hepatica dextra which was in communication with the small bowel. The successful treatment was performed by embolizing the aneurysma.

**Conclusion:**

The reasons for gastrointestinal bleedings are manifold. This case presents a seldom cause of a gastrointestinal bleeding due to an aneurysma of the hepatic arteria. The successful embolization was performed to ultimately stop the bleeding.

## Background

Massive upper gastrointestinal hemorrhage can be the dominant symptom of esophageal varices. Ulcerations of the upper gastrointestinal tract can be alternative reasons for a hemorrhage. Even postoperative complications can lead to extensive bleedings. But the combination of an ulceration and a postoperative complication due to a laparoscopic cholecystectomy is a rare event.

Here we report on a patient with massive bleeding caused by an aneurysma spurium of the arteria hepatica dextra which was induced by a laparoscopic cholecystectomy. Embolization of the arteria was performed and the patient was successfully treated.

## Case presentation

The first hospitalization was due to colicky pain in recent weeks. The clinical presentation showed a patient with recurrent pain in the right upper quadrant of the abdomen. Due to the medical history of the patient with prior gastric cancer who underwent a Roux-en-Y gastric bypass four years ago the ultrasound of the liver and the gallbladder was quite difficult. No additional data were available at the time of admission. A MRCP was performed. The findings of the MRCP were a chronic cholecystitis with multiple stones in the gallbladder and a thickened wall. The laparoscopical ablation of the gallbladder was performed without complication and the patient was dismissed six days later.

**Figure 1:**
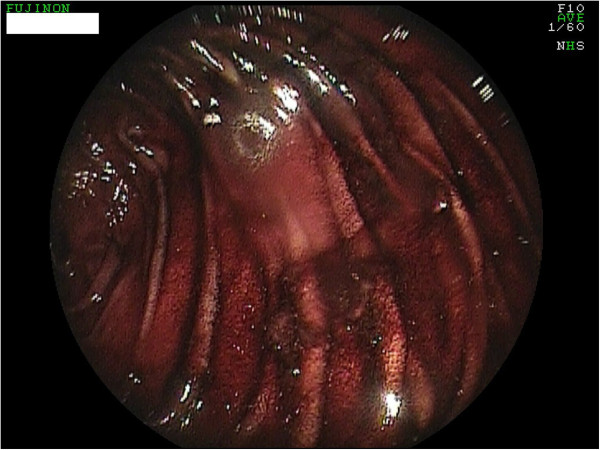
Residues of blood in the jejunum.

**Figure 2:**
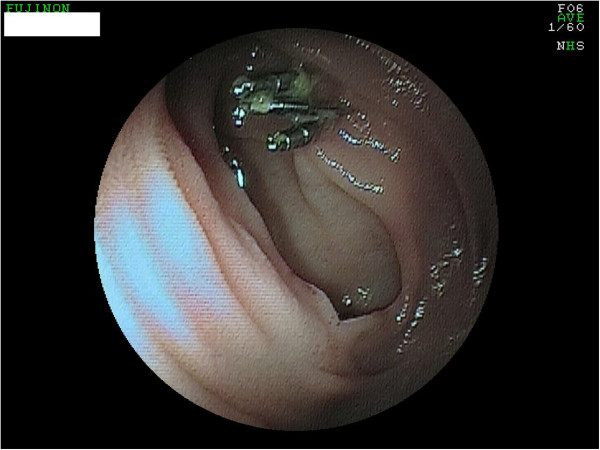
**Ulcus Dieulafoy after clipping.** Photo taken two days after emergency gastroscopy.

At the next hospitalization four months later an upper gastrointestinal bleeding with melena, dizziness and sweating were observed. The initial hemoglobin was 9.3 g/dl and the immediately performed gastroscopies showed extensive residual amounts of blood in the jejunum and an ulcus Dieulafoy. This was successfully addressed with ethoxysclerol (0.5%) and with three clips (Figures 1 and 2). The patient was transferred to the intensive care unit and was monitored for seven days. After the hemoglobin was stable and no further bleeding was apparent the patient left the hospital after eleven days against medical advice.

Two days after dismissal the patient was re-admitted to the emergency unit with melena and in poor condition. We performed a gastroscopy, a capsule endoscopy, ultrasound of the abdomen and CT scans of the abdomen.

**Figure 3:**
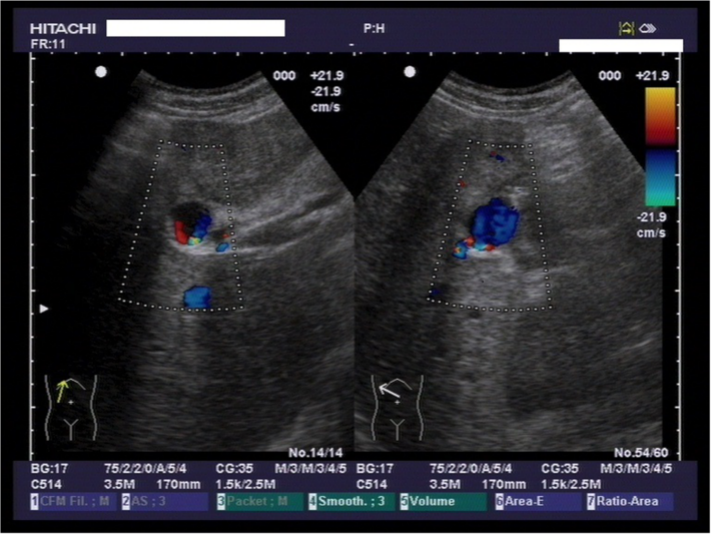
**Doppler ultrasound scan of the liver.** The aneurysma spurium is coloured in the center of each picture.

**Figure 4:**
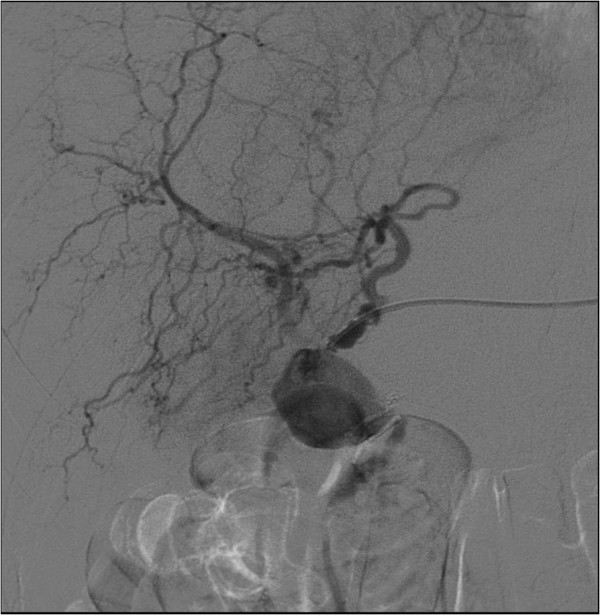
Angiographic ct-scan of the aneurysma before intervention.

**Figure 5:**
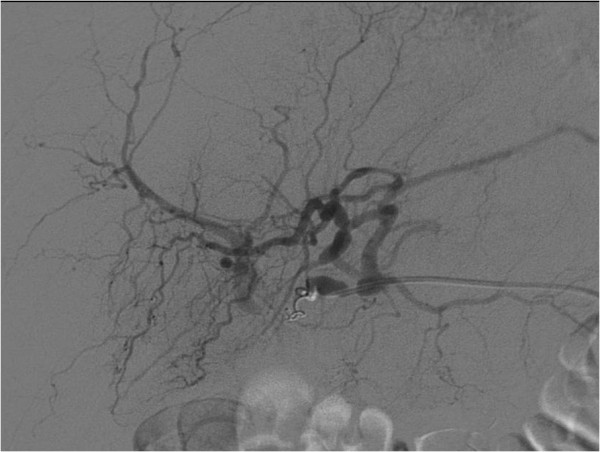
Angiographic ct-scan during intervention.

It was the Doppler ultrasound scan that gave the essential clue towards the origin of the gastrointestinal bleeding (Figure 3). The unusual vascularisation of the arteria hepatica, that was of course the aneurysma spurium, led us to perform an angiographic CT scan (Figure 4). The contrast medium was leaving the lumina at the aneurysma spurium. The patient had developed an aneurysma spurium of the arteria hepatica dextra after laparoscopic cholecystectomy which was in communication with the small bowel. Eventually, this uncommon condition, the aneurysma spurium of the arteria hepatica dextra, was coiled by the interventional radiologist (Figure 5).

## Conclusion

There are many reasons for upper gastrointestinal bleedings (UGB). Their common theme is blood loss proximal to the ligament of Treitz. It is currently accepted that the most frequent reason of an UGB is the ulcus duodeni or ventriculi. It is linked to ~50% of all UGB. It is also referred to as peptic ulcer disease. The other ~50% are divided into esophageal and gastric varices, erosions, Mallory-Weiss syndromes, tumors and uncommon reasons like angiodysplasia (e.g. exulceratio simplex Dieulafoy), blood loss after surgery or trauma. In few isolated cases the reason of blood loss is still unclear or undefined.

Especially in our case we combine two uncommon causes of an UGB. First an exulceratio simplex Dieulafoy (managed by clipping) and second an aneurysma spurium of the arteria hepatica dextra with communication to the small bowel in a patient who underwent a laparoscopic cholecystectomy and a Roux-en-Y gastrectomy.

In the literature you can find an UGB, e.g. haemobilia after laparoscopic cholecystectomy [1], percutaneous liver biopsy [2], trauma, gallstones and cholecystitis [3], biliary drainage, transhepatic cholangiography and tumors [4]. If the reason is an arterial aneurysma, the first line therapy in the management of the aneurysma should be the embolization of the malformed artery. Another treatment possibility is the partial liver resection [5] or, if failed, an open surgery liver resection. In our case we performed a successful interventional embolization of the aneurysm.

A non iatrogenic genesis of hepatic artery aneurysms is reported by Pollono et al. [6]. The case described a patient with three episodes of gastrointestinal bleeding without endoscopic evidence of lesions in the gastrointestinal tract. The patient suffered from systemic lupus erythematosus and systemic vasculitis with inflammation of the hepatic arteries leading to multiple hepatic artery aneurysms. After embolization of the hepatic artery aneurysms no further gastrointestinal blood loss occurred [6].

Uncommon causes of UGB are becoming more frequent in recent years. In databases you observe a slight increase of cases that are dealing with hemobilia and detected aneurysms and pseudoaneurysms. The reason for this may be the high standard of diagnostics in general, the “evolution” of therapeutic approaches, and the possibility to manage adverse events.

### Consent

Written informed consent was obtained from the patient for publication of this Case report and any accompanying images. A copy of the written consent is available for review by the Editor of this journal.

## Competing interest

The authors declare that they have no competing interests.

## Authors’ contributions

OK serves as the lead and corresponding author and drafted the manuscript. BK, AS and TS performed the endoscopic examinations. All authors read and approved the final manuscript.

## Authors’ information

All authors belong (TS, BK) / belonged (OK, AS) to the Department of Internal Medicine II, Witten/Herdecke University, Faculty of Health – School of Medicine, Campus Cologne-Merheim, Germany. OK moved to the Department of Internal Medicine, HELIOS Klinik Wipperfuerth, Germany. AS moved to the Department of Internal Medicine, Gastroenterology and Diabetology, Vivantes Humboldt Klinikum, Berlin, Germany.

## Pre-publication history

The pre-publication history for this paper can be accessed here:

http://www.biomedcentral.com/1471-230X/13/121/prepub
